# Effects of co-habitation between *Anopheles gambiae *s.s. and *Culex quinquefasciatus *aquatic stages on life history traits

**DOI:** 10.1186/1756-3305-5-33

**Published:** 2012-02-09

**Authors:** Eliningaya J Kweka, Goufa Zhou, Leila B Beilhe, Amruta Dixit, Yaw Afrane, Thomas M Gilbreath, Stephen Munga, Mramba Nyindo, Andrew K Githeko, Guiyun Yan

**Affiliations:** 1 Centre for Global Health Research, Kenya Medical Research Institute, P. O. Box 1578, Kisumu 40100, Kenya; 2 Kilimanjaro Christian Medical College, Tumaini University, P. O. Box 2240, Moshi, Tanzania; 3 Program in Public Health, University of California, Irvine, CA 92697, USA; 4 Ecology and Evolutionary Biology, University of California, Irvine, CA 92697, USA

## Abstract

**Background:**

The effective measures for the control of malaria and filariasis vectors can be achieved by targeting immature stages of anopheline and culicine mosquitoes in productive habitat. To design this strategy, the mechanisms (like biotic interactions with conspecifc and heterospecific larvae) regulating mosquito aquatic stages survivorship, development time and the size of emerging adults should be understood. This study explored the effect of co-habitation between *An. gambiae *s.s. and *Cx. quinquefasciatus *on different life history traits of both species under different densities and constant food supply in the habitats of the same size under semi-natural conditions.

**Methods:**

Experiments were set up with three combinations; *Cx. quinquefasciatus *alone (single species treatment), *An. gambiae *s.s. alone (single species treatment); and *An. gambiae *s.s. with *Cx. quiquefasciatus *(co-habitation treatment) in different densities in semi field situation.

**Results:**

The effect of co-habitation of *An. gambiae *s.s. and *Cx. quinquefasciatus *was found to principally affect three parameters. The wing-lengths (a proxy measure of body size) of *An. gambiae *s.s. in co-habitation treatments were significantly shorter in both females and males than in *An. gambiae *s.s single species treatments. In *Cx. quinquefasciatus*, no significant differences in wing-length were observed between the single species and co-habitation treatments. Daily survival rates were not significantly different between co-habitation and single species treatments for both *An. gambiae *s.s. and *Cx. quinquefasciatus*. Developmental time was found to be significantly different with single species treatments developing better than co-habitation treatments. Sex ratio was found to be significantly different from the proportion of 0.5 among single and co-habitation treatments species at different densities. Single species treatments had more males than females emerging while in co-habitation treatments more females emerged than males. In this study, there was no significant competitive survival advantage in co-habitation.

**Conclusion:**

These results suggest that co-habitation of *An. gambiae *s.s. and *Cx. quinquefasciatus *in semi-natural conditions affect mostly *An. gambiae *s.s. body size. Hence, more has to be understood on the effects of co-habitation of *An. gambiae *s.s. and *Cx. quinquefasciatus *in a natural ecology and its possible consequences in malaria and filariasis epidemiology.

## Background

Mosquito breeding habitats vary due to multiple factors such as physico-chemical characteristics of habitats, habitat type and size and predator abundance [1-6]. In African mosquito ecology, immature stages of *Anopheles gambiae *s.s. and *Culex quinquefasciatus *are frequently found to co-occur in diverse habitats such as drainage ditches, swamps and abandoned goldmines [7-11]. In mosquito species, such as *An. gambiae, An. stephensi, Aedes aegypti*, and *Cx. pipiens*, the effects of both abiotic and biotic factors on immatures have been documented to have an influence on life history traits and subsequent adult fitness [12,13].

In population and community ecology, predation, density (experienced mainly under intraspecific interaction), interspecific interactions, and nutrient dependencies are the main mechanisms that regulate population dynamics [3,14,15]. Mosquito populations respond to unfavorable conditions with a drop in one or more vital rates, usually in growth rate and survivorship, but also in fecundity and recruitment [16]. In *Ae. aegypti *and *Ae. albopictus*, density dependence is generally a major component of larval mortality [16]. In these species, density dependence is mostly driven by exploitation competition rather than interference competition [17,18]. Improving the knowledge on density dependence is crucial in determining the ultimate outcome of larval control strategies. Interspecific competition can be a major determinant of species distribution and ultimately of community structure, this phenomenon is widespread among insects [19-21]. Interspecific competition that can be investigated with co-habitation species could lead in some cases to competitive displacement of local population [20,21]; based upon certain ecological principles that state that different species cannot simultaneously occupy the same niche.

Some studies investigating coexistence of the different species under laboratory conditions revealed the existence of predation and cannibalism between species. In *An. gambiae *s.s. (Gilles) and *An. quadrannulatus *(Theobald) co-occurrence, fourth instar larvae of both species were predacious and cannibalistic towards the first and second instar larvae [22]. In another study with *An. arabiensis *(Patton) and *An. gambiae *(Gilles) larvae, cannibalism and predation occurred as a result of maximum interaction in small aquatic habitats and not due to food deprivation [23]. Muturi *et al. *found that predation and cannibalism in co-habitation of *An. gambiae *s.s. and *Cx. quinquefaciatus *happened only between first and third instars of *An. gambiae *and *Cx. quinquefasciatus *when they shared the same habitat [5]. There was no predation effect observed in larvae of *An. gambiae *s.s. and *Cx. quinquefasciatus *of the same age structure [5]. Predatory behaviour has also been found in other species. For example, *Toxorhynchites spp*. have been shown to prey on different mosquito species [24-27]. *Culex (Lutzia) fuscanus *(Wiedemann) were predators of several mosquito species such as *An. stephensi *[28], *Ae. albopictus*, and *Cx. quinquefasciatus *[29].

In previous studies on *Cx. pipiens *and *An. gambiae *s.l., the effect of the factors cited above affecting mosquito dynamics have been widely investigated and little attention has been given to the effect on life history traits such as survivorship, developmental time, sex ratio and wing-length [14,15,30-32]. Predation, coexistence and interspecific competition between *An. gambiae *s.s. and *Cx. quinquefasciatus *larvae may lead to survival rate reduction, developmental time increase, sex ratio distortion and body size reduction which might be a factor altering the fitness of emerging adults and the disease-transmitting ability of one or both species [33-35]. Examination of the wing length variation in the natural population of malaria vectors have been conducted elsewhere [33-36]; but the mosquito species composition was not considered to underscore the observed effect on adult wing-length. The effects of co-habitation between *An. gambiae *s.s. and *Cx. quinquefasciatus *are not clearly known when the larvae experience different densities in a habitat containing the same amount of resources.

Therefore, the objectives of this study were to investigate effects of co-habitation between *An. gambiae *s.s. and *Cx. quinquefasciatus *on different life history traits in semi-natural microcosm experiments. We investigated the downstream effects on survivorship, wing-length, development time, and sex ratio in co-habitation species.

## Methods

### Mosquito collection and rearing

*An. gambiae *s.s. gravid females were aspirated indoors from Iguhu village in the western Kenya highlands. The gravid females were reared singly in a paper cup covered with netting material placed in an insectary maintained at 28.4 ± 1°C and relative humidity of 70 to 80%. The light regime was L 12: D 12. These females were provided with sugar solution (10% sucrose). Eggs laid were collected on wet filter papers and kept in an incubator for 48 hours before hatching. After egg-laying, all females of *An. gambiae *s.l. were taken for polymerase chain reaction to confirm species identification as *An. gambiae *s.s., as described by Scott *et al.*, [37]. The eggs of non *An. gambiae *s.s. species were not used in these experiments. *Cx. quinquefasciatus *egg rafts were collected from septic tanks and polluted abandoned goldmines. Both *An. gambiae *s.s. eggs and *Cx. quinquefasciatus *egg rafts were hatched at the same time and larvae of the same age-structure were used in the experimental set up.

### Artificial habitat preparation (Microcosms) and larvae daily monitoring

Microcosms were made up using washing basins (diameter: 35 centimeters and depth: 15 centimeters) filled with 2 kilogrammes of soil and 3000 milliliters of rain water. These microcosms were covered with mosquito nets to prevent oviposition by other wild gravid mosquitoes. These microcosms were exposed to sunlight as found in natural habitats. Mosquito species composition in microcosms were made up of three larvae compositions i) *An. gambiae *s.s. alone (single species treatment), ii) *An. gambiae *s.s. and *Cx. quinquefasciatus *together (co-habitation treatment), and iii) *Cx. quinquefasciatus *alone (single treatment). These combinations were evaluated in the density of 20, 40, 60, 80 and 100 larvae. For the combined species (*An. gambiae *s.s. and *Cx. quinquefasciatus*), densities were made up of 50% from each species. Every density for each species and composition had ten replicates.

Age structures of surviving mosquito larvae were assessed daily; alive and dead larvae were recorded. Pupae collected from the microcosms were held in paper cups for adult emergence. When pupation started, microcosms were visited twice a day, at 8 am and 5 pm daily for pupae collections.

Competitive advantage of the species was calculated by subtracting the total number of surviving *Cx. quinquefasciatus *or *An. gambiae *s.s. emerged adults from the total number of surviving *Cx. quinquefasciatus *and *An. gambiae *s.s. adults and dividing that quantity by the initial number in each cohort of both species (n = 10, 20, 30, 40, 50).

### Wing-length measurement

Emergent adults were stored with silica gel until subsequent wing-length measurements. The right wing was removed and its length from the arculus to the tip (excluding the fringe) was measured using a scaled microscope. Wing-length was used as a measure of the body size because it has a high correlation to dry body weight [34,38,39].

### Data analysis

The Wilcoxon signed ranks test was used to compare the daily survival rates between species in co-occurrence and same species occurred alone.

The effect of co-occurrence in wing-length was analyzed using Tukey HSD test of one way analysis of variance (ANOVA) to ascertain the effect of co-occurrence on wing-length by sex and species of mosquitoes.

Mean pupation time and female and male emergence proportions were compared between co-occurrence treatment species and single species treatment species using chi-square test.

The sex ratio was calculated as the number of emerged adult females divided by the total number of emerged adults (both males and females) for each density and species. The sex ratio deviation from 0.5 within a species was calculated using test of equal proportion, Fisher's exact test.

The competitive survival advantage analysis was computed using one way analysis of variance (ANOVA) by comparing the number of adult mosquitoes emerged between the co-habitation of *An. gambiae *s.s. and *Cx. quinquefasciastus*.

## Results

### Effect of co-occurrence on survivorship

**Figure 1:**
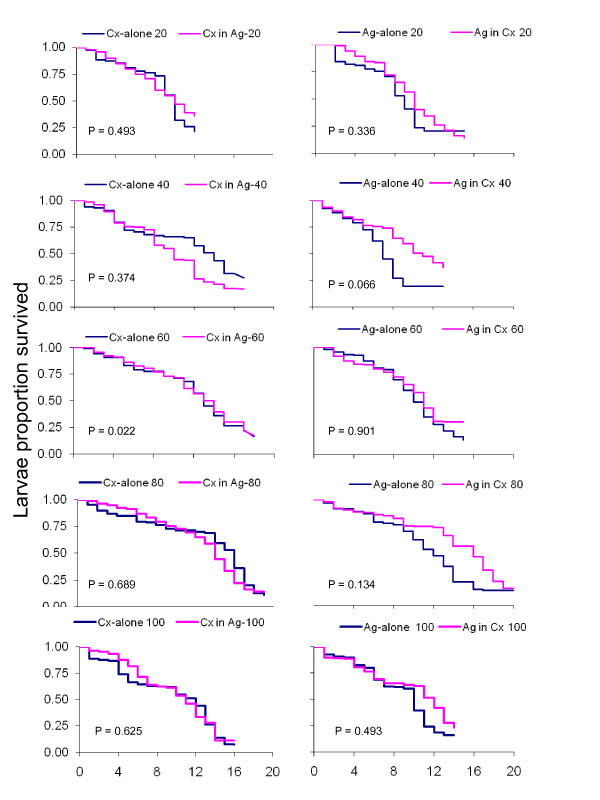
**The comparison of daily survival rates between *An. gambiae *s.s (Ag) and *Cx. quinquefasciatus *(Cx) occurred alone and that co occurred**.

No effect of co-occurrence on daily survival rate was observed for either species. There was no significant difference between the survivorship in single and co-occurring treatments for all densities (Figure 1). Only at a density of 60 was there more *Cx. quinquefasciatus *that survived in a single species treatment than in co-occurrence treatment (Figure 1). All larvae of each species were scored either alive or dead and none were lost during counting.

### Developmental Time

**Figure 2:**
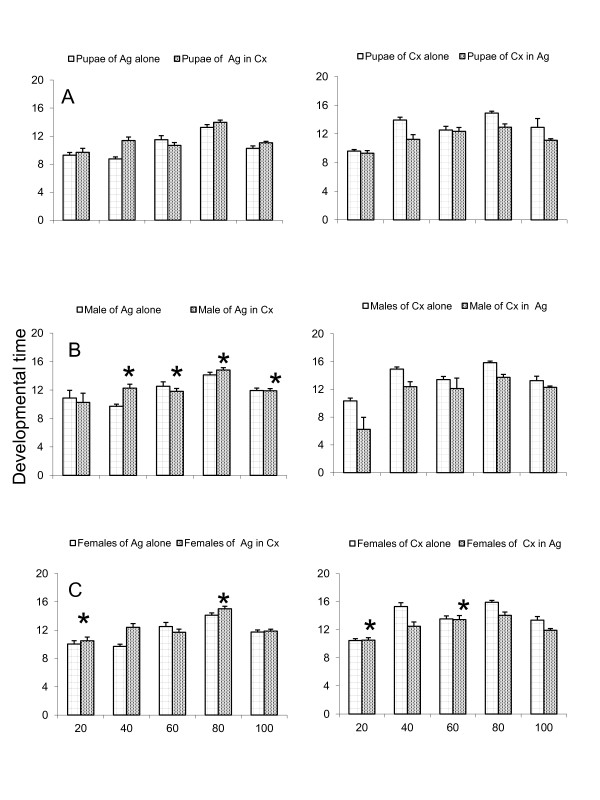
**Mean developmental time of pupae (A), males (B) and female (C) emerged of *An. gambiae *s.s (Ag) and *Cx. quinquefasciatus *(Cx) in single species treatment and in co-habitation treatment**. (**Note**: Asterisk (*) in graph demotes the groups which do not differ statistically significantly).

There were significant differences in the pupae developmental time for each species at all densities with the single treatment developmental time being shorter than the co-habitation treatments (Figure 2A). The developmental time for male emergence for *Cx. quinquefasciatus *was significantly higher in single than in co-habitation treatments at a density of 20 only; for *An. gambiae *s.s., only the densities of 60 (P = 0.121) and 80 (P = 0.213) were not significantly different between single and co-habitation treatments co-habitation (Figure 2B). The developmental time for females *Cx. quinquefasciatus *emergence in single treatment was significantly shorter than the co-habitation treatment in all densities except in larvae density of 20 (P = 0.721) and 60 (P = 0.441); for *An. gambiae *s.s., single treatments were significantly shorter at all densities except 20 (P = 0.081) and 80 (P = 0.067) where there was no difference between single and co-habitation treatments (Figure 2C).

### Wing-length measurements

**Figure 3:**
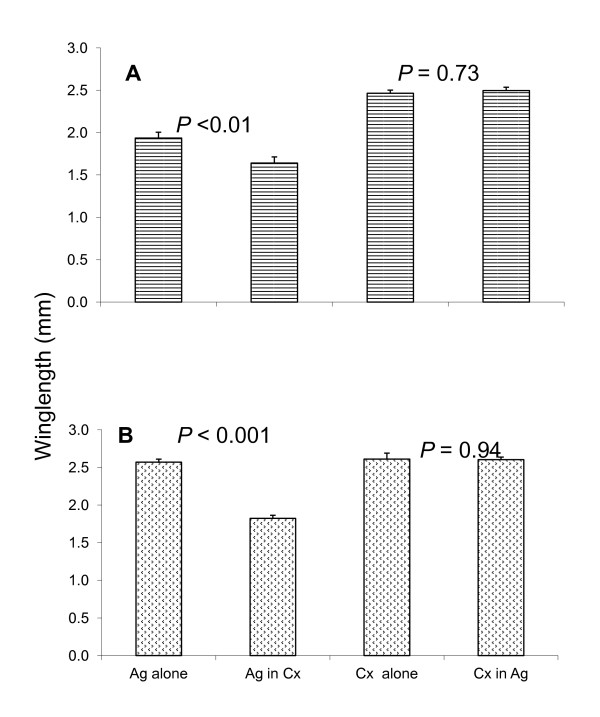
**Mean wing-length differences between males (A) and females (B) of *An. gambiae *s.s (Ag) and *Cx. quinquefasciatus *(Cx) in single species treatment and in co-habitation treatment**.

The overall co-occurrence significantly affected wing-length for both females and males of *An. gambiae *s.s. as the mean wing-length in co-occuring treatment is significantly lower than in single species treatment. Co-occurrence treatment had no significant effect on mean wing-length for female and male mosquitoes of *Cx. quinquefasciatus *(Figure 3).

### Sex ratio

The sex ratio among single species treatment of *An. gambiae *s.s. in all larval densities was significantly lower than 0.5 and the same tendency was found in *Cx. quinquefasciatus*, which favoured males in both species (Table 1). The sex ratio among co-habitation *An. gambiae *s.s. and *Cx. quinquefasciatus *was significantly different from 0.5 at densities of 20, 40 and 80 for *An. gambiae *s.s. and at densities of 20, 40, 60, and 80 for *Cx. quinquefasciatus *(Table 2). In these cases, the sex ratio was significantly higher than 0.5, favouring females.

**Table 1:** Sex ratio for each species in different densities among single species treatments

Density	*An. gambiae s.s*	*Cx. quinquefasciatus*
20	43.2 (111)*	37.1(132)*

40	33.1(260)*	26.1(253)**

60	13.9 (397)**	13.0 (377)**

80	41.4 (399)*	34.2 (427)**

100	44.2 (77)*	44.7 (472)*

**Note**: Sex ratios significantly different from 0.5 are indicated by *. (* P < 0.05, **P < 0.01). Sex ratio was calculated from the number of individuals that emerged (indicated in brackets).

**Table 2:** Sex ratio for each species in different densities among co-habitation treatments

Density	*An. gambiae *s.s	*Cx. quinquefasciatus*
20	64.6 (96)*	74.5 (55)**

40	66.1 (109)*	63.7 (124)*

60	59.1 (215)	66.2 (195)**

80	66.3 (261)**	67.5 (277)**

100	62.2 (172)	52.5 (204)

**Note**: Sex ratio significantly different from 0.5 are indicated by *. (* P < 0.05, **P < 0.01). Sex ratio was calculated from the number of individuals that emerged (indicated in brackets).

### Competitive advantage

At each density (i.e. 20, 40, 60, 80 and 100), there was no significant difference in the survival advantage shown by any species over another in co-habitation treatments. Also, in overall analysis, there was no significant competitive survival advantage for any species in co-habitation treatments as the mean advantage was around zero (DF = 1, F = 3.7749, P = 0.058).

## Discussion

The findings of this study have demonstrated that when there is co-occurrence between *An. gambiae *s.s. and *Cx. quinquefasciatus *in habitats with different larval densities, there are variations in different life traits. For *An. gambiae *s.s. in co-habitation treatments, both males and females had reduced body size compared to *An. gambiae *s.s. in single species treatment. The sex ratio of both *An. gambiae *s.s. and *Cx. quiquefasciatus *have demonstrated a natural composition of having more males than females in single species treatment while the co-habitation treatments had more females emerging than males. Daily survival rates of *An. gambiae *s.s. in single and co-habitation treatments had no significant difference at any density while Cx*. quiquefasciatus *had similar results except at a density of 60 larvae. In developmental time, more pupae and adults were observed to emerge earlier from single species treatment than in co-habitation treatments. None of the species found were shown to have survival advantage against each other in co-habitation treatments. This study had similar and different findings compared to studies carried out elsewhere with these species of larvae co-habiting or when crowded [5,6,22,23,40-42]. Similar phenomenon of co-habitation was found to have effects within *Aedes aegypti *population [43,44]. *An. gambiae *s.s. in co-habitation treatments had no significant difference with the *An. gambiae *s.s. in single species treatment in daily survival rates which presumably shows that the habitats' resources and survivorship were dependants, and also the density used might not been enough to have the impact clearly observed in those artificial habitats resources. However, the size of the habitats and amount of food was determined to be sufficient, therefore, there was no significant difference observed in daily survivorship as outcome of resources constraints (Figure 1). In other studies, as the density of larvae increases while the amount of food and space maintained at a constant level, the survivorship was higher when the larval density was lower [14,40]. However, this was not the case in our study results. In co-habitation treatments, *Cx. quinquefasciatus *fed on lower micro layer surfaces while *An. gambiae *s.s. had more food sources on upper surface micro layers [14,40]; the difference is due to the fact that the two species have different micro layers feeding preferences in their habitats, resulting in different food sources [29,31]. This reveals that in species competition, when food resources and space are kept constant and larval density increase in the same species as dependant variable in limiting the survivorship of aquatic stages of mosquitoes [11,15,30,31,41,44,45].

The aggregation of larvae of different species tend to produce a growth retardation chemical which limits the growth of competing species when existing in large number in a habitat [46]. In our study, the increase of larvae in both single and co-occurrence treatments had no survivorship difference but the developmental time decreases as the density increase in habitats. A similar effect was observed in studies conducted elsewhere [46].

The developmental time for *An. gambiae *pupae and the sex ratio of the emerged adults were found to be species and density dependant (Figure 2). The lack of significant differences in mortalities in higher densities for co-habitation treatments have shown that food sources and space in habitats had no effect on higher densities in our experiments as observed in other laboratory and field observational studies [29,31]. In the single species treatments, the sex ratio for both *An. gambiae *s.s. and *Cx. quinquefasciatus *was significantly lower than 0.5 and varied with density. This indicated that males were in higher proportions than females in single species treatments. The sex ratio among the emerged mosquitoes of each species at each density for co-habitation treatments was significantly different with higher proportion from 0.5 and varied with density in each species. This indicates that among the emerging mosquitoes, the highest proportion consisted of females rather than males. In previous studies, the adult sex ratio of female to male was 1:3 for *An. gambiae *s.s. [7] and 1:2 for *Cx. quinquefasciatus *[47]. Other observational studies done in field situations reported skewed female to male ratios ranging from 1:10 up to 1:600 during swarming [38,48,49]. This study has reported for the first time the effect of larvae in experimental co-habitation consisting of more females than males. These findings might have an epidemiological impact in natural populations in that, having more females than males will increase the biting rates and higher probabilities of disease transmissions; however, having fewer males in a population might cause lower fecundity and reproductive rates for female mosquitoes.

The co-habitation of *An. gambiae *s.s. and *Cx. quinquefasciatus *exists in nature [7,8,10,11]. In this study it was revealed that the co-habitation mostly affects *An. gambiae *s.s. by reducing the wing-length size which is a proxy measure of the body size and the ability of host seeking and fecundity for female mosquitoes [34,50,51]. Large-bodied *Aedes triseriatus *females have been associated with increased parity rates (which are a measure of blood-feeding behaviour) among collected female mosquitoes [52,53]. Similar results were reported by Nasci [53,54] for *Psorophora columbiae *(Dyar and Knab), *Aedes vexans *(Meigen) and *Aedes aegypti *(L.). Female mosquitoes that have emerged with a small body size have reduced blood meal succession hence lower fecundity and survivorship and subsequently, low parasite transmission efficiency [34,36,55-57]. In other studies, laboratory experiments have shown that female malaria vectors with small body size feed more frequently, hence this influences arbovirus transmission. For males, the body size affects flight ability which translates into reduced swarming efficiency [42,56] as small males refrain from swarming for longer or begin to swarm earlier. This also means that they are less successful at mating than larger males [42,58]. Similar findings have been observed in *An. gambiae *s.s. [41,42].

The ecological life history of mosquitoes is affected mostly by larval density and food sources in habitats [31,59]. Availability of food resources in habitats determines the number of adults emerging [41], their body size [34,59], and their survivorship [41]. Crowded larvae are at a disadvantage because they are faced with greater inter and intra-specific competition for food resources and are therefore, at a risk for reduced survival [41]. They are also exposed to higher levels of toxic waste products, crowding chemicals and physical interference from other larvae [41,60,61].

The competitive advantage in species was not observed to be a factor of concern in this study. Competitive displacement is based upon the ecological principle that different species sharing the same trophic level can inhibit each other from occupying the same niche [62,63]. Hardin [64] found that this competitive principle in various laboratory studies showed a population competition in which one of the competing species would theoretically become extinct. In *An. gambiae *s.s. and *Cx.quinquefasciatus *co-habitation species, neither was found to have the competitive advantage over the other. This implies that between the two species (*An. gambiae *s.s. and *Cx. quinquefasciatus*), neither can lead to extinction of the other in the conditions tested. In Australia, it was found that introduction of *Aedes notoscriptus *had led to the extinction *Ae. aegypti *[65].

## Conclusion

These study findings suggest that the co-habitation treatment can have considerable effect on body size of *An. gambiae *s.s. and sex ratio of emerging adults. There is a need to explore the observed effect in natural conditions and estimate its epidemiological significance in malaria and filariasis transmission reduction and control.

## Conflict of interest

The authors declare that they have no competing interests.

## Authors' contributions

EJK conceived, designed, implemented the study and supervised experiments. EJK, LB and GZ did data analysis and interpretation. EJK wrote the manuscript. EJK, GZ, LB, AD, AY, TMG, SM, MN, AKG and GY revised the manuscript. All authors approved the final version for submission.
